# Psychosocial risk factors associated with social anxiety, depressive and disordered eating symptoms during COVID-19

**DOI:** 10.3934/publichealth.2023003

**Published:** 2023-02-07

**Authors:** Carlee Bellapigna, Zornitsa Kalibatseva

**Affiliations:** School of Social and Behavioral Sciences, Stockton University, USA

**Keywords:** COVID-19, risk factors, mental health, social anxiety, depression, disordered eating

## Abstract

The coronavirus (COVID-19) pandemic has disrupted society and negatively impacted mental health. Various psychosocial risk factors have been exacerbated during the pandemic, leading to the worsening of psychological distress. Specifically, a need for structure, loneliness, concerns about body image and social media use are risk factors previously implicated in poor mental health. The current study examines how these risk factors are associated with mental health outcomes (i.e., social anxiety, depressive and disordered eating symptoms) during the COVID-19 pandemic (January–March 2021). A total of 239 participants were recruited (average age = 24.74, 79% female, 68% White). The results revealed that a need for structure, loneliness and social media use were positively associated with social anxiety. In addition, loneliness, negative concerns about body image and social media use were significantly related to disordered eating and depressive symptoms. Lastly, when examined all together, the overall model for risk factors predicting mental health outcomes was significant: Wilks' Λ = 0.464, *F*(12, 608.814) = 17.081, *p* < 0.001. Loneliness and social media use were consistently associated with all psychological symptoms. These results emphasize the need for interventions for social anxiety, depressive and disordered eating symptoms that encourage structured daily activities, social connection, positive perception of oneself and mindful social media use.

## 1. Introduction

The coronavirus disease (COVID-19) has disrupted society and significantly impacted people's lives, placing a strain on physical and mental health [1]. In 2020 and 2021, the guidelines advised to limit social contact to prevent spreading the disease. Separation from loved ones and the disruption of daily routines (e.g., attending school/work in person, eating, socializing) led to isolation and loneliness [2]. Both are risk factors for depression and anxiety [3],[4]. In addition, social media use and preoccupation with one's body also increased during the COVID-19 pandemic [5]. Both constructs have been related to psychological distress and disordered eating [6]. The present study aims to examine how psychosocial risk factors during the COVID-19 pandemic (January 2021 through March 2021), such as the disruption of routine and structure, loneliness, concerns about body image and social media use, are associated with symptoms of social anxiety, depression and disordered eating.

### 1.1. Psychological distress: Social anxiety, depression and disordered eating

Research has demonstrated significant adverse mental health consequences from the effects of the COVID-19 pandemic [7]–[9]. Multiple risk factors were exacerbated, and negative mental health outcomes emerged. Some of the disruptions that influenced mental health in the first year of the pandemic have included changes in routine and structure, loneliness, concerns about body image and social media use. Because these risk factors have been found to independently contribute to psychological distress [10]–[13], it is likely that they would have an impact on individuals' mental health when experienced at the same time during the unique circumstances of the COVID-19 pandemic. Based on these assumptions, the present study aimed to examine the relationship between common risk factors experienced during the COVID-19 pandemic and mental health outcomes. Some of the most pressing concerns that have been previously associated with these risk factors include depression, social anxiety and disordered eating, which have shared comorbidities [14],[15].

Various studies found an increase in depressive symptoms and major depressive disorder (MDD) in college students and the general population during the COVID-19 pandemic [16],[17]. MDD is a common mood disorder that affects how a person feels, thinks and manages daily tasks or activities [18]. Symptoms of depression include a depressed mood, lack of interest or pleasure, significant weight and appetite changes, insomnia or hypersomnia, psychomotor agitation or retardation, fatigue or loss of energy, feelings of worthlessness or inappropriate guilt, diminished ability to think or concentrate; and recurrent thoughts of death or suicidal ideation [19]. Hawes and colleagues [20] found that the rates of clinical depression increased nearly three times in female participants during the pandemic. This may be related to a greater concern about being confined at home [20]. As compared to men [19], women and girls experience higher rates of internalizing issues such as anxiety and depression, along with disordered eating, which refers to maladaptive eating behaviors that do not meet the criteria for an eating disorder [19],[21]. Engaging in disordered eating behaviors increases the risk for significant physical and mental stress. Some disordered eating behaviors include frequent dieting, anxiety toward specific foods or meal skipping; preoccupation with weight and food and concerns about body image; feelings of guilt or shame associated with eating; and chronic weight fluctuations [19]. Ramalho and colleagues [22] reported high rates of disordered eating behaviors during the COVID-19 lockdown in a Portuguese sample. Psychological distress fully mediated the association between the COVID-19 pandemic impact and disordered eating.

Research suggests that disordered eating behaviors and increased social media use may have played a large role in the ability to cope with stressors during the COVID-19 pandemic, including changes to the home environment and adjusting to disruptions in normal daily activities [7],[22]–[24]. These disruptions in daily life also contributed to maintain symptoms of social anxiety, which have been found to remain high in the context of COVID-19 [25]. Social anxiety, or social phobia, is described as an excessive or maladaptive reaction to social situations when a person is exposed to unfamiliar people or situations that could make them feel embarrassed or humiliated [26]. Individuals with social anxiety worry that they will be negatively evaluated and avoid social situations. Due to COVID-19, many face-to-face interactions were reduced or eliminated to protect physical health. As a result of changing norms and social expectations (e.g., greetings, social distancing), social anxiety may increase in ambiguous situations [27],[28]. Research suggests that the COVID-19 pandemic has contributed to increases in social anxiety and depression among adolescents and young adults [20]. Numerous studies have suggested that precautionary measures that were implemented to prevent the spreading of the disease affected feelings of isolation and loneliness [29], which are risk factors for depression and anxiety disorders [2],[25]. The disruption of routine, loneliness, concerns about body image, increased social media use and negative affectivity, which have been exacerbated during the COVID-19 pandemic, are potential risk factors for depression, social anxiety and disordered eating [21],[24]. No studies have looked at all three of these mental health outcomes together before or after the pandemic. By examining the effects of the COVID-19 pandemic on depression, social anxiety and disordered eating, we consider the implications of the discussed risk factors for future mental health interventions.

### 1.2. Psychosocial risk factors

#### 1.2.1. Need for structure

Having a routine helps establish structure in an individual's life. Structure is a way to simplify the world into a manageable form, and it may involve establishing routines, reducing the amount of information requiring attention and utilizing a social script when interacting with others [30]. The COVID-19 pandemic has placed restrictions on daily activities and limited access to places that may assist with emotional regulation (e.g., participating in sports, visiting a therapist) for individuals [24]. The disruption of physical activity and exercise was associated with elevated levels of depression [31]. The constraints placed on regular activity and the disruption of routine may lead to heightened body image concerns and disrupted eating patterns [24],[32]. In addition, concerns about the disruption of routine positively predicted depressive symptoms [5]. Studies have also found a significant relationship between a personal need for structure and anxiety [10],[30]. As the COVID-19 pandemic disrupted regular routine for many individuals, this study examines the need for structure as a risk factor for social anxiety, depressive and disordered eating symptoms.

#### 1.2.2. Loneliness

Another prominent risk factor for psychological distress during the COVID-19 pandemic is loneliness. Cacioppo et al. [11] described the feeling of loneliness as a discrepancy between the social relations an individual prefers and actual social experience. Although prevention measures, such as social distancing and quarantine, were necessary to prevent the spread of the disease, they have been associated with increased levels of loneliness and social isolation [4],[29]. Several studies previously reported loneliness to be a risk factor for depressive symptoms, social anxiety symptoms and disordered eating behaviors [29],[33],[34]. To further the existing research, this study examines loneliness as a risk factor for all three outcomes in the context of the pandemic.

#### 1.2.3. Body image

Body dissatisfaction is another risk factor for disordered eating, depressive and social anxiety symptoms [21],[22],[35]. Body image, or body apperception, is a multifaceted construct that refers to the attitudes toward one's body and actions to obtain what one perceives as ideal, and it is usually conceptualized as the evaluation of attractiveness and feelings associated with body shape and size [5]. Sociocultural factors, such as media use, family or peer influence and sports participation can impact body concerns, along with one's gender [32],[36]. Individuals who reported more positive body attitudes experienced greater subjective happiness [37]. Conversely, negative perceptions, feelings and thoughts of one's body are linked to disordered eating behaviors, which are often comorbid with anxiety and depressive symptoms [12],[22],[38]. Aderka and colleagues [35] also found that symptoms of social anxiety disorder were significantly associated with disturbances in body image and attitudes toward one's appearance. An increase in video-chat use during the COVID-19 pandemic heightened exposure to self-image, which may be related to appearance-related body image [5].

#### 1.2.4. Social media use

With increased social distancing and limited face-to-face interaction during the COVID-19 pandemic, many daily activities, including school and work, were modified to fit an online format. Social media use was on the rise before the COVID-19 pandemic [13]; it further increased during the pandemic, and thus may pose as a risk factor for poor mental health outcomes [7]. While limited social media use is often viewed as a positive way to stay connected and interact with others, heavy social media use has been associated with negative psychological well-being [13],[39]. Sherman and colleagues [40] found that the use of social media to determine appropriate or acceptable social behavior provides quantifiable measures of peer endorsements, which could impact mental health and concerns about body image. Social media use during the COVID-19 pandemic and body dissatisfaction could predict disordered eating patterns and concerns related to weight outcomes, including dieting, weight gain and dietary restraints [24]. Therefore, it is important to examine the relationship between social media use and psychological distress during the pandemic.

#### 1.2.5. Rationale and hypotheses

**Figure 1.**
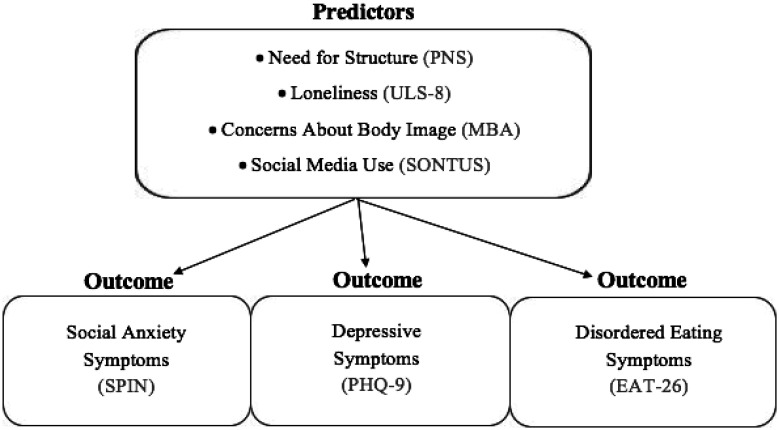
Proposed model of COVID-19 risk factors and outcomes.

The present study examines the need for structure, loneliness, concerns about body image and social media use (as risk factors) and social anxiety, depressive and disordered eating symptoms (as outcomes; see Figure 1) during the COVID-19 pandemic. The global pandemic has highlighted the importance of social connection, support and structure, which have all been disrupted by COVID-19. No previous study has examined these predictors and outcomes together or in the context of the pandemic. This study has important implications about understanding the relationship between risk factors experienced during the COVID-19 pandemic and symptoms of social anxiety, depression and disordered eating. Based on the existing research, the study's hypotheses were as follows:

1. Need for structure, loneliness, concerns about body image and social media use will be associated with social anxiety symptoms.
2. Need for structure, loneliness, concerns about body image and social media use will be associated with depressive symptoms.
3. Need for structure, loneliness, concerns about body image and social media use will be associated with disordered eating symptoms.
4. Need for structure, loneliness, concerns about body image and social media use will be associated with disordered eating symptoms, social anxiety symptoms and depressive symptoms together.

## 2. Materials and methods

### 2.1. Participants

There were 239 participants in this study, of which 19.7% (*n* = 47) were male, 79.1% (*n* = 189) were female and 1.3% (*n* = 3) were non-binary or non-conforming. In this sample, 6.7% of individuals identified as Black or African American (*n* = 16), 10% identified as Hispanic (*n* = 24), 5.9% identified as Asian (*n* = 14), 67.8% identified as White (*n* = 162), 9.2% identified as Multiracial/Biracial (*n* = 22) and 0.4% identified as other (*n* = 1). The majority (83.3%, *n* = 199) of participants were current college students and 16.7% were not (*n* = 40). Of those who were college students, 98.5% were students at a midsize public northeastern university (*n* = 195). The average age of the participants in the sample was M = 24.74 (*SD* = 11.14). Table 1 provides complete demographic information.

**Table 1.** Demographics.

	*n*	%
**Gender**	239	100
Female	189	79.1
Male	47	19.7
Non-Binary	3	1.3
**Race**	239	100
Black/African American	16	6.7
Hispanic	24	10.0
Asian	14	5.9
White	162	67.8
Native American/Pacific Islander	1	0.4
Multiracial/Biracial	22	9.2
**Education Level**	238	99.6
High School Degree or Equivalent	61	25.6
Some College	110	46.2
2-Year Degree	24	10.1
Bachelor's Degree	28	11.8
Master's Degree	8	3.4
Doctorate	5	2.1
Other	2	0.8
**College Student**	239	100
Current college student	199	83.3
Not a college student	40	16.7
**Class Standing**	198	82.8
First-Year	75	37.9
Sophomore	42	21.2
Junior	41	20.7
Senior	36	18.2
Graduate Student	3	1.5
Other	1	0.4
**Depression Diagnosis**	239	100
Have been diagnosed	49	20.5
Have not been diagnosed	190	79.5
**Anxiety Disorder Diagnosis**	239	100
Have been diagnosed	67	28.0
Have not been diagnosed	172	72.0
**Eating Disorder Diagnosis**	239	100
Have been diagnosed	15	6.3
Have not been diagnosed	224	93.7

### 2.2. Measures

#### 2.2.1. Demographic questionnaire

Demographic questions included information about the participants' age, gender, race/ethnicity, education, place of residence and mental health history.

#### 2.2.2. COVID-19 effects and experiences

Participants were asked a series of questions about the effects of COVID-19 on their lives. Examples include: “In the past 6 months, how much has the COVID-19 pandemic affected your: Daily Routine; Loneliness; Body Image; Social Media Use (Facebook, Twitter, Instagram, etc.)?”; and “Related to the COVID-19 pandemic, in the past 6 months, have you experienced any of the following: Feelings of anxiety; Feelings of depression; Changes in eating patterns (i.e., frequent dieting; feelings of guilt associated with eating; preoccupation with weight, food or body image)?”. Responses were recorded on a 5-point Likert scale (1 = Not at All to 5 = Extremely). Questions were also intended to prime participants to think in the context of the COVID-19 pandemic.

#### 2.2.3. Personal need for structure scale

The Personal Need for Structure (PNS) scale [41] is a 12-point instrument used to assess an individual's preference for a known structure and familiar situations with three sub-scales: preference for orderliness, discomfort with unpredictability and disdain for ambiguity. Responses were recorded on a 6-point Likert scale (1 = Strongly Disagree to 6 = Strongly Agree). Statements on the PNS scale include “[i]t upsets me to go into a situation without knowing what I can expect from it”, “I like being spontaneous” (reverse-coded) and “I hate to change my plans at the last minute”. Four of the scale items were reverse-coded. Higher scores indicate a stronger need for structure and less flexibility and adaptability when it comes to unpredictable situations [41]. The PNS scale demonstrated good internal consistency in the original study (Cronbach's *α* = 0.84) [41] and acceptable internal consistency (Cronbach's α = 0.74) in this study.

#### 2.2.4. UCLA loneliness scale

The UCLA Loneliness Scale (ULS-8) [42] was designed to measure an individual's feelings of loneliness and social isolation. The eight-item measure consists of responses on a 4-point Likert scale (1 = Often to 4 = Never), with each item rated as either “I often feel this way”, “I sometimes feel this way”, “I rarely feel this way” and “I never feel this way”. Higher scores indicate lower degrees of loneliness. The measure was not recoded to maintain its original format. The ULS-8 demonstrated good internal consistency in the original study (Cronbach's *α* = 0.84) [42] and in this study (Cronbach's α = 0.83).

#### 2.2.5. Measure of body apperception scale

Originally developed for breast cancer patients, the Measure of Body Apperception (MBA) scale is an eight-item instrument that measures investment in—or concerns about—one's body image [43]. Examples of items include “[i]t's important to me to look my best all the time”, and “I feel good about myself only if I know I look good to other people”. Participants responded on a 6-point Likert scale how much they agree or disagree with each statement (1 = Strongly Disagree to 6 = Strongly Agree). Higher scores indicate more concern about body image, or a more negative perception of oneself. The MBA scale demonstrated acceptable internal consistency in the original study (Cronbach's *α* = 0.78 for physical appearance and *α* = 0.53 for body integrity) [43] and in this study (Cronbach's α = 0.74 for physical appearance and Cronbach's α = 0.60 for body integrity). The total score was used to measure concerns about body image in this study (Cronbach's α = 0.77).

#### 2.2.6. Social networking time use scale

Although it is a relatively new scale, the social networking time use scale (SONTUS) [37] is a standardized instrument that is used to measure the amount of time spent on social networking sites. An adapted version of the SONTUS was used to reduce time for completion and to include items relevant to the COVID-19 climate. The abbreviated questionnaire consisted of 20 items on a 6-point Likert scale (1 = Always to 6 = Never). Participants were asked to indicate how often they used social networking sites like Facebook, Instagram, Twitter, Snapchat, TikTok, etc., during the past week in a set of situations and places. Examples of statements included “When you are at home sitting idly”; “When you are watching TV, news, sports, etc.”; “When you are in bed about to sleep”; “When you are receiving a class lecture”; “When you need to reduce your emotional stress”; and “When you are online doing school or job-related work”. Participants answered questions related to the following: relaxation and free periods, academic-related periods, stress-related periods and motives for use. Lower scores indicated higher social media use. The original SONTUS demonstrated excellent internal consistency (Cronbach's *α* = 0.92) [44]. The abbreviated version in this study also showed excellent internal consistency (Cronbach's α = 0.95).

#### 2.2.7. Eating attitudes test

The Eating Attitudes Test (EAT-26) is a 26-item screening measure used to determine if an individual is experiencing abnormal eating behaviors [45]. EAT-26 was not designed as a diagnostic tool for eating disorders. Responses are on a 6-point Likert scale about symptoms occurring within the last 6 months (Always = 3, Usually = 2, Often = 1, Sometimes = 0, Rarely = 0, Never = 0). Higher scores (above 20) indicate disordered eating behaviors. Sample items on the EAT-26 include “[I] [a]m terrified about being overweight”, “[I] [f]eel extremely guilty after eating” and “[I am] [a]ware of the calorie content of foods that I eat”. The EAT-26 demonstrated excellent internal consistency before (Cronbach's *α* = 0.90) [45] and good internal consistency in this study (Cronbach's α = 0.83).

#### 2.2.8. Social phobia inventory

The Social Phobia Inventory (SPIN) [46] is a 17-item, self-reported measure used to evaluate an individual's fear, avoidance and physiological discomfort in social situations (i.e., social events, being criticized, talking to strangers). Responses are on a 5-point Likert scale from 0 (Not at all) to 4 (Extremely), and they are about symptoms occurring in the last week. The assessment screens for social phobia and includes statements such as “I avoid talking to people I don't know” and “I am afraid of doing things when people might be watching”. The highest possible score is 68, and a cut-off value of 19 may be used to differentiate participants with and without social phobia. Higher scores indicate more symptoms of social anxiety. Social anxiety symptoms are reflected through different levels of scores for the SPIN, which demonstrated excellent internal consistency in the original study (Cronbach's *α* = 0.94) [46] and in this study (Cronbach's α = 0.93).

#### 2.2.9. Patient health questionnaire

The Patient Health Questionnaire (PHQ-9) is a nine-item depression module used to measure and screen for depressive symptoms [47]. Participants are asked about depressive symptoms experienced within the last 2 weeks, and responses are on a 4-point Likert scale (Not at all = 0, Several days = 1, More than half the days = 2, Nearly every day = 3). Examples of statements on the PHQ-9 are as follows: “Little interest or pleasure in doing things” and “Feeling tired or having little energy”. Scores can range from 0 to 27. Scores below 10 indicate no depressive symptoms, and higher scores suggest more severe depressive symptoms, which may require further evaluation for diagnosis. The PHQ-9 demonstrated excellent internal consistency in the original study (Cronbach's *α* = 0.89) [47] and in this study (Cronbach's α = 0.91).

### 2.3. Procedure

Participants were primarily recruited through the university psychology participant pool (SONA) and granted class credit as an incentive to participate. They signed a consent form and completed the survey through Qualtrics. This study was approved by the university's Institutional Review Board.

Additional recruitment methods focused on recruiting individuals through social media venues (i.e., Facebook, Twitter and Instagram) and inviting members of student groups via email to participate in the study. Anyone over the age of 18 was eligible to participate. Participants who completed the study outside of SONA were invited to be entered into a raffle to win one of 30 $10 Amazon gift cards. The two incentives could not be combined. The participants were able to enter the raffle on a page separate from their completed responses. Their identity was not connected to their responses. All data were collected between January and March of 2021 when social distancing and mask wearing were required locally at the university and nationally.

### 2.4. Ethics approval of research

The study was approved by Stockton University Institutional Review Board (IRB#: 2020.206). Further, informed consent was obtained via the online consent form from the respondents before participating in the study.

## 3. Results

All analyses were conducted by using IBM SPSS Statistics 26. Table 2 shows results about pandemic perceptions. The means, standard deviations and Pearson *r* correlations for all predictors (need for structure, loneliness, perceived concerns about body image and media use) and outcomes (social anxiety, depressive and disordered eating) appear in Table 3. All of the risk factors had statistically significant correlations with all outcomes.

**Table 2.** COVID-19 pandemic effects (in the past 6 months, N = 239).

	Not at all	A little bit	Somewhat	Very much	Extremely
Daily routine	2.9%	10%	23%	38.1%	25.9%
Loneliness	11.3%	13.4%	29.7%	28%	17.6%
Body image	18%	17.6%	25.5%	22.6%	16.3%
Social media use	13.4%	6.3%	15.5%	27.6%	37.2%
Feelings of anxiety	12.6%	11.7%	20.9%	25.1%	29.7%
Feelings of depression	15.5%	19.2%	25.9%	17.6%	21.8%
Changes in eating patterns	18%	18.8%	17.2%	23%	23%

**Table 3.** Descriptive statistics and correlations.

	M (SD)	**1**	**2** ^a^	**3**	**4** ^a^	**5**	**6**	**7**
1. Personal Need for Structure (PNS)	48.11 (7.53)	1	−0.263**	0.331**	−0.134*	0.385**	0.225**	0.209*
2. Loneliness (ULS)^a^	21.51 (4.9)		1	−0.256**	0.299**	−0.536*	−0.574**	−0.271**
3. Concerns About Body Image (MBA)	22.58 (6.85)			1	−0.283**	0.290**	0.308**	0.360**
4. Social Media Use (SONTUS)^a^	62.49 (21.71)				1	−0.309**	−0.339*	−0.283**
5. Social Anxiety Symptoms (SPIN)	24.5 (14.55)					1	0.541*	0.320**
6. Depressive Symptoms (PHQ-9)	8.05 (6.62)						1	0.420**
7. Disordered Eating Symptoms (EAT-26)	10.97 (9.99)							1

Note: *p ≤ 0.05 level (2-tailed); **p ≤ 0.01 level (2-tailed); ^a^High scores of ULS and SONTUS indicate low loneliness and social media use, respectively.

### 3.1. Hypothesis 1

A multiple regression analysis showed that the need for structure (*β* = 0.239, *p* < 0.001), loneliness (*β* = −0.416, *p* < 0.001) and social media use (*β* = −0.134, *p* = 0.017) were significant predictors for social anxiety symptoms. Concern about body image was not a significant predictor (*β* = 0.063, *p* = 0.275). Overall, the model accounted for 37.4% of the variance in social anxiety symptoms: *R^2^* = 0.374, *F*(4, 232) = 34.725, *p* < 0.001.

### 3.2. Hypothesis 2

A multiple regression analysis revealed that loneliness (*β* = −0.485, *p* < 0.001), concerns about body image (*β* = 0.123, *p* = 0.033) and social media use (*β* = −0.154, *p* = 0.006) were significant predictors for depressive symptoms. The need for structure was not a significant predictor (*β* = 0.039, *p* = 0.488). Overall, the model accounted for 37.6% of the variance in depressive symptoms: *R^2^* = 0.376, *F*(4, 233) = 35.160, *p* < 0.001.

### 3.3. Hypothesis 3

A third multiple regression analysis indicated that loneliness (*β* = −0.138, *p* = 0.033), concerns about body image (*β* = 0.253, *p* < 0.001) and social media use (*β* = −0.161, *p* = 0.012) were significant predictors for disordered eating symptoms. The need for structure was not a significant predictor (*β* = 0.07, *p* = 0.278). Overall, the model accounted for 18.7% of the variance in disordered eating symptoms: *R^2^* = 0.187, *F*(4, 233) = 13.440, *p* < 0.001.

### 3.4. Hypothesis 4

A multivariate multiple regression using the multivariate general linear model function in SPSS was conducted, and the overall model for risk factors predicting mental health outcomes was significant: Wilks' Λ = 0.464, *F*(12, 608.814) = 17.081, *p* < 0.001.

## 4. Discussion

The present study contributes to the existing research on mental health during the COVID-19 pandemic by examining mental health risk factors from our everyday lives that were exacerbated during this time. The study's hypotheses were largely supported, as greater scores for the need for structure, loneliness, concerns about body image and social media use were associated with more social anxiety, depressive and disordered eating symptoms. When examined together, all psychosocial COVID-19-specific risk factors were related to all mental health outcomes.

Participants also self-reported their perceptions of changes in daily routine, loneliness, concerns about body image and social media use during the COVID-19 pandemic. Overwhelmingly, responses indicated that, in the past 6 months, people had felt less connected, used more social media, experienced more disruption in schedules and had more disturbances in body image compared to pre-pandemic levels. Participants reported the most changes in their daily routines (87%), followed by social media use (80.3%), loneliness (75.3%) and body image (64.4%).

Individuals who needed more structure, felt lonely and used social media more often had higher social anxiety symptoms. These findings are consistent with previous research, which established a relationship between each risk factor and social anxiety [10],[33]. Despite having a moderate correlation with social anxiety symptoms (*r* = 0.29, *p* < 0.01), concern about body image was not associated with social anxiety symptoms when the other risk factors were included. This finding is inconsistent with previous research that found that reduced satisfaction of appearance and reduced feelings of attractiveness were significantly associated with social anxiety symptoms [35]. One possible explanation for this finding is the reduced social interaction during the pandemic. During the COVID-19 restrictions, one's concerns about body image may have been less associated with social anxiety due to less in-person contact [48]. This result may also be related to the measure we used to assess concerns about body image, as it focused more on concerns about physical appearance and body integrity, which may differ from traditional measures of body satisfaction.

Our findings are also consistent with previous research [10],[30], which demonstrated that individuals with stronger preference for structure in their lives may experience more social anxiety symptoms. However, a personal need for structure was not significantly associated with depressive or disordered eating symptoms. This finding is inconsistent with Choukas-Bradley and colleagues' study [5], which found that concerns about changes to appearance through disruptions in routine (e.g., gym closures) were associated with increased depressive symptoms during the COVID-19 lockdown. While having a moderate correlation with depressive and disordered eating symptoms on its own (*r* = 0.26, *p* < 0.01; *r* = 0.21, *p* < 0.01, respectively), the need for structure did not predict depressive or disordered eating symptoms when all other risk factors were included. Rodgers et al. proposed that the disruption of routine and structure during the COVID-19 pandemic may lead to heightened concern about body image as well as disrupted eating patterns, which are often comorbid with depressive symptoms [32]. However, the current study did not find support for this proposed theoretical model. Our findings were also inconsistent with research before and during the COVID-19 pandemic [22],[49], which suggested that, with constraints placed on regular activity, changes to one's routine and structure may lead to heightened concern about body image, as well as disrupted eating patterns. On the contrary, people who valued more structure did not report higher disordered eating symptoms in this study. Although this finding was unexpected, one possible explanation is that individuals learned to adapt and developed their own routines after several months into the pandemic, as data for this study were collected in January–March 2021.

Our study provided further support that loneliness during COVID-19 was a substantial risk factor for psychological distress [29],[33],[34]. Loneliness is more likely in individuals experiencing social isolation or separation from their friends or family [50]. People who reported higher perceived loneliness also had elevated social anxiety, depressive and disordered eating symptoms. Physical distancing guidelines and self-isolation mandates during the COVID-19 pandemic were associated with increased social disconnection and self-reported loneliness [4],[29]. Among college students, feelings of loneliness may be intensified by being unable to attend in-person classes, visit friends, have social gatherings or attend club meetings. The observed effect sizes of loneliness as a risk factor were large, suggesting that, on a population level, it has a meaningful impact. Therefore, reducing levels of loneliness is an important implication of this study for prevention and intervention programs, as it may lower risk for social anxiety, depressive and disordered eating symptoms.

Social media use was also a risk factor significantly associated with social anxiety, depressive and disordered eating symptoms during the COVID-19 pandemic. This is consistent with Fernández-Aranda and colleagues' proposed model [24], which suggested that more time spent using social media (as observed during the COVID-19 pandemic) would be a potential risk factor for disordered eating. Increased exposure to unrealistic or unattainable bodies or lifestyles, which are usually highlighted on social media, may explain negative mental health outcomes. Yet, social media also provided support and connection during COVID-19 for users who shared experiences or offered accountability [6].

### 4.1. Clinical implications

Although we collected data during the COVID-19 pandemic (January–March 2021), our findings have implications beyond it, as the psychosocial risk factors we examined exist outside of the pandemic and were particularly exacerbated under the strict public health measures. Our findings emphasize that loneliness during periods of social isolation contributed the most to psychological distress. Therefore, we need to encourage belongingness, maintain social connections in a safe way and reach out for support during periods of required self-isolation [51]. At the same time, the Centers for Disease Control and Prevention (CDC) [51] recommended reducing social media use and limiting phone, television and computer screen time during idle periods. Too much or too little social media use may be associated with worse mental health [13],[38]. Helping individuals develop a schedule to balance their daily social media use and avoid excessive use may decrease the risk of psychological distress. Remaining socially connected in moderation may also help to lessen feelings of loneliness, which lower the risk for social anxiety, depressive and disordered symptoms [6],[29]. In addition, individuals may also benefit from regular physical activity and the implementation of modifiable coping strategies, such as spending time with family or talking with friends online, to support mental health during the COVID-19 pandemic [8]. Mental health care providers may recommend their clients to develop structure in daily activities, take mindful breaks, focus on positive aspects of their bodies and balance social media use in order to mitigate negative feelings and psychological distress. This may be particularly important for people who are working/studying remotely after the pandemic and have few in-person interactions. Although the initial COVID-19 lockdowns are behind us, there may be other pandemics or natural disasters in the future that would require people to shelter in place. The lessons we have learned from our past experiences and this study are to decrease loneliness by increasing social connection among our social networks through a variety of media (e.g., phone, video chat) while not overly relying on social media.

### 4.2. Strengths, limitations and future directions

The study empirically tested a model of risk factors for social anxiety, depressive and disordered eating symptoms during the COVID-19 pandemic. Despite its strong theoretical and methodological rationale, the study had some limitations. First, the data are cross-sectional and cannot establish cause and effect. Therefore, the findings of this study only show associations. Second, our sample had limited diversity in terms of age, gender and race. The sample included participants with a mean age of 24.74 years and mostly consisted of college students (>80%) and individuals who identified as women (>75%). In addition, 67.8% of the sample consisted of non-Hispanic White individuals. Thus, the generalizability of our findings may be limited. Furthermore, this study relied on self-reported measures about symptoms, which are not diagnostic. Longitudinal studies would be able to provide more information about temporal relationships between risk factors and outcomes. Additionally, future research studies need to include more representative samples of various age groups, genders, races and ethnicities. It is important to examine the continuing effects of the COVID-19 pandemic on mental health after vaccination and the removal of risk mitigation measures. Although many people in the USA have “returned to normal”, challenges to mental health remain in the presence of high rates of depression and anxiety.

## 5. Conclusions

This study provided support that lacking structure, increased loneliness, negative cocerns about body image and frequent social media use in the context of the COVID-19 pandemic were associated with poorer mental health. The results point to the need for interventions for social anxiety, depression and disordered eating that facilitate structure and routines, social connection, positive perception of oneself, and mindful social media use. These findings emphasize the importance of openly discussing challenges during the COVID-19 pandemic and beyond in order to reduce stigma and improve mental health. Although the course of the pandemic has changed since March 2020 and the president of the USA announced the end of the pandemic in September 2022, there are still health concerns related to COVID-19. Some people are still struggling with balancing their physical and mental health needs and figuring out their “new normal” related to work, relationships and perception of oneself. Future research needs to develop and test specific prevention and intervention programs that help increase connectedness and maintain balanced social media use to improve mental health during periods that may require self-isolation and beyond the COVID-19 pandemic.
